# Double-balloon enteroscopy for luminal evaluation of the excluded stomach: a retrospective multicenter analysis

**DOI:** 10.1016/j.igie.2025.12.012

**Published:** 2026-01-13

**Authors:** Peter Albares, F. Otis Stephen, Lauren Pioppo-Phelan, Catherine Hudson, Alice Parish, Iris Vance, Jason Stibbe, Donna Niedzwiecki, Daniel Wild

**Affiliations:** 1 Department of Internal Medicine, Duke University School of Medicine, Durham, North Carolina, USA; 2 Division of Gastroenterology & Hepatology, Cedars Sinai Medical Center, Los Angeles, California, USA; 3 Division of Digestive Diseases, The University of California, Los Angeles, California, USA; 4 Division of Gastroenterology, Duke University School of Medicine, Durham, North Carolina, USA; 5 Section of Gastroenterology, Louisiana State University Health Sciences Center, New Orleans, Louisiana, USA; 6 Department of Biostatistics and Bioinformatics, Duke University School of Medicine, Durham, North Carolina, USA

## Abstract

**Background and Aims:**

After Roux-en-Y gastric bypass (RYGB), accessing the excluded or remnant stomach (RS) is difficult using standard endoscopy, and double-balloon enteroscopy (DBE) is often used when pathology is suspected. Data on the use of DBE for this purpose are limited. We investigated the success rate, technical factors, and findings when using DBE to access the RS after RYGB.

**Methods:**

This was a retrospective analysis of adult patients with RYGB who underwent DBE to access the RS between January 2018 and July 2023 across 3 academic medical centers. Primary aims were identifying the technical success rate of accessing the RS and associated endoscopic findings. Secondary aims were identifying patient characteristics, procedure indications, factors behind technical failures, studies preceding DBE, endoscopic therapies performed, and procedural adverse events.

**Results:**

Eighty-nine patients underwent DBE to access the RS after RYGB for anemia/bleeding (56.2%), abdominal pain (32.6%), and abnormal imaging (44.9%). The RS was successfully accessed in 71.9% of cases. The most common reasons the RS was not accessed were proximal pathology (40%), anastomotic angulation (32%), and limb length (16%). Many (40.4%) examination results were normal. The most common findings were inflammatory (30.3%) or vascular (15.7%), 6.7% had polyps, and 5.6% had malignant neoplasm. Biopsy (50.6%) and hemostatic maneuvers (20.2%) were commonly performed. Accessing the RS was not associated with patient age, procedural indication, or time from RYGB. DBE served as definitive management in 29.2% of cases, and no procedural adverse events occurred.

**Conclusions:**

The RS in RYGB can be successfully and safely accessed with DBE.

## Background and Aims

**Supplementary Figure 1:**
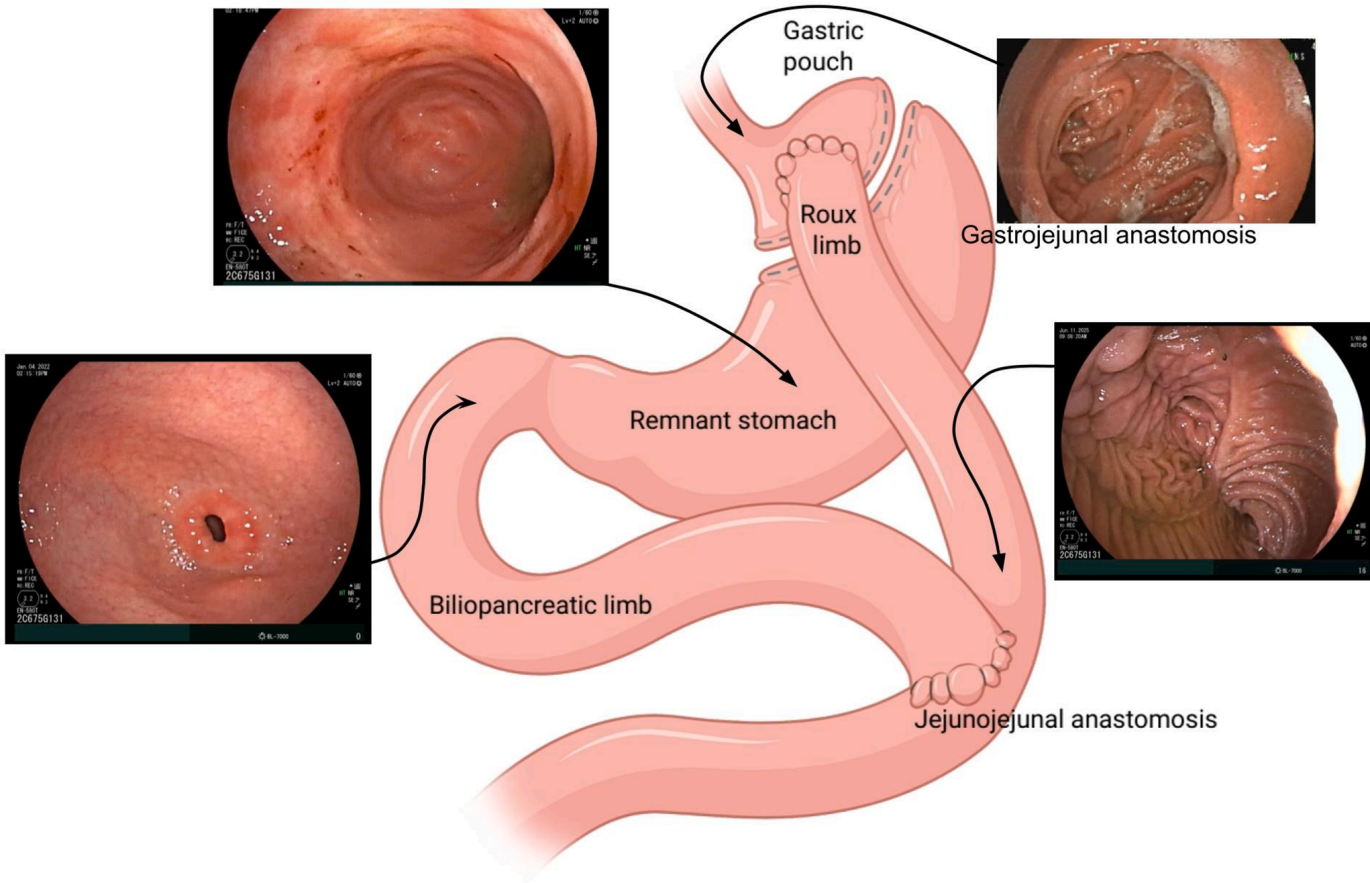
Altered gastrointestinal anatomy after Roux-en-Y gastric bypass (RYGB). Representative endoscopic images demonstrate the post–RYGB anatomy and key landmarks. The small gastric pouch is shown with the gastrojejunal anastomosis leading into the alimentary (Roux) limb. Additional views illustrate the jejunojejunal anastomotic separation of the biliopancreatic limb from the alimentary limb, the pylorus from the biliopancreatic limb, and the excluded (remnant) stomach, which is not in continuity with the alimentary tract. Endoscopic orientation and labeled landmarks highlight the surgically altered pathways relevant to diagnostic and therapeutic endoscopy.

Bariatric surgery is increasingly common in the United States. After gastric sleeves, Roux-en-Y gastric bypass (RYGB) is the second most commonly performed procedure with over 62,000 performed in 2022.^1^ After RYGB, the excluded or remnant stomach (RS) and proximal duodenum with its adjacent biliary structures become inaccessible to standard endoscopy due to their distance from the mouth (and anus) and the typically sharp angle the biliary limb takes from the jejunojejunal anastomosis (Supplementary Fig. 1, available online at www.igiejournal.org). This makes it challenging to evaluate suspected luminal pathology in these areas when symptoms or abnormal findings on cross-sectional imaging arise. The challenge of accessing these areas can result in uninvestigated findings or the need for surgical exploration.

Double-balloon enteroscopy (DBE) is a well-described procedure that has been used in clinical practice since the early 2000s.^2^^,^^3^ It offers the ability to gain access to deep segments of the small bowel and, in patients with post-RYGB anatomy, a way to directly evaluate the RS and proximal duodenum. Despite its well-established use for this indication, there are limited data surrounding the use of DBE to specifically evaluate the RS because the majority of existing literature over the last 2 decades has focused on the use of DBE to access the ampulla for endoscopic retrograde cholangiopancreatography (ERCP).^4^, ^5^, ^6^, ^7^, ^8^ A small prospective study from Brazil investigated patients who underwent elective DBE after RYGB and demonstrated that the RS could be endoscopically accessed in approximately half of the cohort with 75% of these patients having abnormal findings within the RS.^9^ Another very small retrospective analysis reviewed patients who experienced acute adverse events involving their RS after RYGB, and 2 of these patients were successfully managed with DBE.^10^ Small studies have reported the characteristics of RS pathology, including metaplasia, ulcers, stenosis, gastritis, and cancer.^11^ Others have demonstrated alterations in RS epithelial characteristics and fluid, which create a proinflammatory and premalignant environment.^9^^,^^12^^,^^13^ Because the number of patients who undergo RYGB increases, so will the need for a safe, effective, and reliable method of examining the RS should pathology arise within it. The purpose of this study is to evaluate the technical success and diagnostic utility of DBE to evaluate the RS in patients with RYGB.

## Methods

This was a retrospective, multicenter study of adults (≥18 years) with a prior RYGB who underwent DBE for luminal evaluation of the RS between January 2018 and July 2023 at 3 academic referral centers across the United States: Duke University Health System (DUHS) (Durham, NC, USA), Louisiana State University Medical Center (LSU) (New Orleans, La, USA), and Cedars-Sinai Medical Center (Los Angeles, Calif, USA). The endoscopists who performed the DBEs were all experienced double-balloon enteroscopists with at least 5 years of DBE experience who perform multiple DBEs weekly and have performed several hundreds of examinations over their careers. All procedures were performed using the EN-480T Balloon Enteroscope (Fujifilm Corp, Tokyo, Japan). The procedures were performed with the patient under general anesthesia or monitored anesthesia care, depending on the medical center and the patient. Patients were placed in either a supine or left-lateral position depending on the preference of the endoscopist. A stiffening wire (Zutron Medical, Lenexa, Kans, USA) was used on demand, and none of the sites routinely performed these examinations under fluoroscopy.

Primary aims included identifying the technical success rate of accessing the RS and the associated endoscopic findings. Secondary aims included identifying patient characteristics, indications for DBE, the relationship between findings and technical success rate and when the RYGB was performed, evaluations before DBE, endoscopic therapies performed, and procedural adverse events.

After attainment of site-specific Institutional Review Board approval, a focused electronic medical record chart review was conducted to identify all patients who underwent DBE to assess the RS after RYGB over the span of years included in our study. Pertinent procedural (indication, procedure year, procedure duration, whether the RS was accessed, reason the RS was not accessed, endoscopic findings, interventions performed, and adverse events), clinical (personal and family history of gastrointestinal (GI) malignancy, evaluations performed before DBE, postdiagnostic management, and pathology results), and demographic (patient age, sex, and year of RYGB) data were collected and stored in a shared, secure REDCap database (Atlantic Beach, Fla, USA) hosted at Duke University.^14^

Demographic, clinical, and procedural characteristics were summarized overall using frequency and percent for categorical characteristics, and median and interquartile range (IQR) for continuous characteristics. To assess the secondary associations of interest, we evaluated a Wilcoxon rank sum test, *χ*^2^ test, or Fisher exact test as appropriate. No adjustments were made for multiple testing. All analyses were conducted using SAS software, version 9.4 (IBM Corp, Armonk, NY, USA).^15^

## Results

A total of 89 patients underwent DBE for luminal evaluation across the 3 institutions over the 5 years included in the study, and each recorded patient underwent only a single DBE. All examinations were performed from an antegrade (peroral) direction, and similar numbers of patients came from all 3 medical centers: 33 (37%) from Cedars-Sinai, 25 (28%) from DUHS, and 31 (35%) from LSU. The median age of our cohort was 55 years (IQR: 48-67), and 80.9% of the patients were female, which is consistent with the known gender distribution in bariatric surgery patients (Table 1).^15^, ^16^, ^17^ Only a small number of patients had a personal (4/88; 4.5%) or family (8/88; 9.1%) history of GI malignancy. Some patients had their RYGB surgically revised, but the date of the original RYGB was used for time duration variables; the median time between the original RYGB and DBE was 13 years (IQR: 5-16).

**Table 1:** Demographics, procedural characteristics, and endoscopic findings

Demographic information	Total n = 89
Age (years), median (IQR)	55 (48-67)
Sex: Female, n (%)	72 (80.9)
Personal history of GI malignancy, n (%)∗	4 (4.5)∗
Family history of GI malignancy, n (%)∗	8 (9.1)∗
Indication for DBE (more than 1 could be indicated), n (%)	
Anemia/bleeding	50 (56.2)
Abnormal imaging (cross-sectional, VCE, and EUS)	40 (44.9)
Abdominal pain	29 (32.6)
Other	9 (10.1)
Time from RYGB to DBE (years), median (IQR)	13 (5-16)
Procedure duration (minutes), median (IQR)	59 (36-86)
Procedural success, n (%)	
RS successfully accessed	64 (71.9)
RS not accessed	25 (28.1)
Reasons for nonaccess are listed below:	
Proximal pathology	10 (40)
Anastomotic angulation	8 (32)
Limb length	4 (16)
Fixation/adhesions	2 (8)
Obstruction	1 (4)
Findings (more than 1 could be indicated), n (%)	
Normal	36 (40.4)
Ulcer	13 (14.6)
Inflammatory	27 (30.3)
Vascular changes	14 (15.7)
Polyp	6 (6.7)
Neoplasm	5 (5.6)
Post-DBE management (more than 1 could be indicated), n (%)	
Definitive endoscopic management	26 (29.2)
Further imaging after DBE	18 (20.2)
Repeat DBE required	17 (19.1)
Referral to surgery	14 (15.7)
Referral to oncology	5 (5.6)
Medical management based on findings	28 (31.5)

*DBE*, Double-balloon enteroscopy; *EUS*, endoscopic ultrasound; *GI*, gastrointestinal; *IQR*, interquartile range; *RS*, remnant stomach; *RYGB*, Roux-en-Y gastric bypass; *VCE*, video capsule endoscopy.

∗ 1 patient missing information, not included in denominator of percentage.

The procedures often had multiple overlapping indications, which most commonly included anemia/bleeding (56.2%), abdominal pain (30.3%), and abnormal imaging findings (including cross-sectional and video capsule endoscopy) (44.9%). Because video capsules rarely visualized the RS, video capsule endoscopy typically prompted these examinations if it revealed bleeding suspected to be potentially coming from the RS or proximal biliary limb. Four patients (4.5%) required surveillance of previously detected abnormalities like adenomas or dysplasia within the RS, 2 (2.2%) underwent the procedure for attempted foreign body removal (in 1 a migrated biliary stent, and a suspected silastic band from a prior bariatric intervention in the other) and 1 patient had it for placement of an enteral feeding tube. Most patients had undergone antecedent endoscopic evaluation with upper endoscopy (75.3%), colonoscopy (60.7%), computerized tomography of the abdomen and pelvis (46.1%), video capsule (42.7%), and/or push enteroscopy (18.0%), but only a few (6.7%) had a previous device-assisted examination (single-balloon enteroscopy, spiral enteroscopy, or previous DBE).

The RS was accessed in 64 (71.9%) patients with a median DBE procedure duration of 59 minutes (IQR: 36-86 minutes). Reasons the RS was not accessed (25 cases) included anastomotic angulation (32%), limb length (16%), and adhesions/fixation (8%). In 10 (40%) patients, the RS was not accessed because significant pathology was detected in the duodenum, obviating the need to maneuver more proximally into the RS. The time between RYGB and DBE was not significantly associated with successful access of the RS (*P* = .54). There were no patient or clinical characteristics, including age, sex, and personal and family history of GI malignancy, or indications that were significantly associated with successful access of the RS (Table 2).

**Table 2:** Characteristics by remnant access success

Characteristics	No access (n = 25)	Successful access (n = 64)	Total (n = 89)	*P* value
Study site	–	–	–	.005∗
Cedars-Sinai	4 (16.0%)	29 (45.3%)	33 (37.1%)	–
Duke	6 (24.0%)	19 (29.7%)	25 (28.1%)	–
LSU	15 (60.0%)	16 (25.0%)	31 (34.8%)	–
Age	–	–	–	.45†
Median	56.0	55.0	55.0	–
IQR	45.0-61.0	48.0-69.0	48.0-67.0	–
Recorded sex	–	–	–	.89∗
Male	5 (20.0%)	12 (18.8%)	17 (19.1%)	–
Female	20 (80.0%)	52 (81.3%)	72 (80.9%)	–
Personal history of GI malignancy	–	–	–	1.00‡
Unknown	1	0	1	–
Yes	1 (4.2%)	3 (4.7%)	4 (4.6%)	–
No	23 (95.8%)	61 (95.3%)	84 (95.5%)	–
Family history of GI malignancy	–	–	–	.85‡
Missing	1	0	1	–
Positive family history	2 (8.3%)	6 (9.4%)	8 (9.1%)	–
Negative family history	19 (79.2%)	46 (71.9%)	65 (73.9%)	–
Unknown/unrecorded	3 (12.5%)	12 (18.8%)	15 (17.1%)	–
Indications for DBE (not mutually exclusive)	–	–	–	–
Anemia/bleeding	14 (56.0%)	36 (56.3%)	50 (56.2%)	.98∗
Abdominal pain	6 (24.0%)	21 (32.8%)	27 (30.3%)	.42∗
Abnormal VCE	6 (24.0%)	14 (21.9%)	20 (22.5%)	1.00‡
Abnormal cross-sectional imaging	4 (16.0%)	17 (26.6%)	21 (23.6%)	.41‡
Time from RYGB to DBE, years	–	–	–	.54†
Missing	2	14	16	
Median	14.0	13.0	13.0	
IQR	6.0-19.0	4.0-16.0	5.0-16.0	

*DBE*, Double-balloon enteroscopy; *GI*, gastrointestinal; *IQR*, interquartile range; *LSU*, Louisiana State University; *RYGB*, Roux-en-Y gastric bypass; *VCE*, video capsule endoscopy.

∗ X^2^.

† Wilcoxon rank sum test.

‡ Fisher exact test.

Thirty-six patients (40.4%) had a normal or unremarkable examination result. Twenty-seven (30.3%) had inflammatory findings, 14 (15.7%) had vascular changes, including friable oozing mucosa, and 13 (14.6%) had ulcer findings. Neoplasm was found in 5 patients (5.6%), 4 (4.49%) with adenocarcinoma, and 1 (1.12%) with a neuroendocrine tumor. All of the patients with adenocarcinoma had abnormal computed tomography scan results, 3 had associated abdominal pain, and 2 had concomitant bleeding. One patient (1.1%) had an advanced adenoma. Of all findings, only adenocarcinoma was significantly associated with time from RYGB to DBE: in patients with adenocarcinoma, there was a median of 22 years between DBE and their original RYGB compared to patients without adenocarcinoma, where the median time was 13 years (*P* = .01, Supplementary Table 1, available online at www.igiejournal.org).

Endoscopic interventions performed included biopsies (50.6%), hemostatic maneuvers (20.2%), dilation (4.5%), and polypectomy (3.4%). One patient underwent stent placement of the pylorus due to malignant outlet obstruction of their RS, and 1 patient had a gastrostomy tube placed into their RS during DBE. Endoscopic management was considered definitive in 26 (29.2%) patients who did not require additional endoscopic procedures, surgical interventions, or additional imaging after their DBE. Eighteen (20.2%) patients had further imaging, 17 (19.1%) required repeat endoscopy, and 14 (15.7%) were referred for surgery. Medical management directed at bleeding or inflammatory findings encountered during DBE was recommended in 28 (31.5%).

## Discussion

Pathology seldom arises in the RS after RYGB, but when it is suspected, direct luminal assessment can be challenging. Because the RS can rarely be reached with standard upper endoscopy or push enteroscopy, DBE is often pursued as a way to directly inspect the RS. However, despite this regularly arising in clinical practice, there are very little data to show how successful, safe, or effective this is because the majority of interest in studying DBE in patients with RYGB has focused on its ability to allow for successful cannulation of the bile ducts for ERCP. Our study, the largest one to investigate this topic (to our knowledge), shows that the RS can be safely accessed with DBE in the majority of patients in whom it is attempted. We suspect that the actual rate of RS access is likely even higher than the 72% we demonstrate because the RS could likely have been accessed in some or all of the 10 patients whose examinations were ended when significant pathology was found in the duodenum.

Given the limits of this retrospective study and the inherent difficulty of determining the reasons for thwarted scope advancement during DBE, it is difficult to know the exact reason for failure to intubate the RS. In general, excessive, unreducible looping and/or fixation and resistance to scope advancement and reduction maneuvers were what thwarted access to the RS. These barriers were suspected to be caused by sharp angulation of the takeoff of the biliary limb from the jejunojejunal anastomosis, the length of the intestinal limbs (not formally measured but suspected in some cases based on the experience of the endoscopist), and/or adhesions (suspected with excess fixation and resistance). Unfortunately, these factors are all difficult to predict or determine before attempting DBE unless operative reports pertaining to the initial RYGB are available, which, in our experience, is rarely the case. Even at referral centers with expertise in DBE, we also suspect that the experience and DBE volume of the provider is a significant factor because the rates of RS access ranged from 88% at Cedars Sinai, the highest volume center in our study, to 51.6% at LSU and the lowest volume site, with a rate of 76% at Duke, where the volume falls between the 2 other centers (Table 2). Although these differences likely primarily reflect varying experience performing DBE, they may also suggest possible differences in how RYGB is performed regionally, but further study would be required to delineate this further. Options for management of cases in which the RS could not be accessed with DBE depend on the nature of the suspected finding and the available expertise of the center involved. Surgically created access via laparoscopy or endoscopic ultrasound–directed transgastric ERCP (EDGE)/gastric access temporary for endoscopy (GATE) are options if the concern for pathology is high. If the examination was prompted primarily by nonspecific findings or symptoms, monitoring with serial imaging may also be appropriate.

Although DBE has traditionally been associated with long procedure times, our examinations were performed in a median of only 1 hour. It is also important to note that no adverse events occurred during these procedures despite the patients' altered anatomy and frequently encountered bowel fixation. Because DBE allows for the performance of the full range of endoscopic therapies, our study showed that it provided definitive management in close to one-third of these patients.

Although these examination results were often normal, inflammatory and vascular findings were common, and advanced adenomas and adenocarcinoma were detected. This study was not powered or designed to calculate a true incidence of disease, but the rate of gastric adenocarcinoma seen in this study, 4.49%, is much higher than either the expected rate of gastric malignancy in patients undergoing endoscopy for dyspeptic symptoms, including alarm symptoms (less than 0.8% in a number of studies featuring Western populations),^18^, ^19^, ^20^ or than the overall incidence of gastric cancer in the United States, which currently accounts for 1.5% of new cancers diagnosed each year.^21^ In a review of the available literature in 2019, there were only 17 reported cases of malignancy arising in the RS, but this may be subject to reporting bias.^22^ Although referral bias may have contributed to our unexpectedly high number of gastric cancers, we feel that these findings should not be overlooked. None of the patients with gastric cancer in this study were known to harbor the traditional risk factors such as genetic predisposition, *Helicobacter pylori* infection, or a history of atrophic gastritis with intestinal metaplasia, and previous studies suggest that gastric bypass surgery itself can result in an increase in chronic inflammation, bile exposure, and chronic stasis in the RS and may be associated with altered gene expression patterns in the RS, which likely increase the risk of malignancy.^9^^,^^12^^,^^13^^,^^23^, ^24^, ^25^ More prolonged exposure to these factors, as well as advancing age, likely explain our finding that increased time from initial RYGB was associated with adenocarcinoma in the RS. Consequently, we think that our findings warrant further exploration.

Because this was not a comparative study, DBE should be considered along with the alternate ways to evaluate the lumen of the RS after RYGB. Laparoscopic access is safe and effective but is associated with longer recovery times and the cost and resources of an operating room.^26^ More recently, EDGE or GATE has been developed as a way to allow for more ready access to the RS or ampulla in RYGB patients.^27^^,^^28^ This procedure has some clear advantages when comparing it to DBE. It also has high technical success rates and, once performed, access to these areas is possible with any foregut endoscope, which allows for the full spectrum of therapies, including biliary interventions, endoscopic ultrasound with fine-needle aspiration, endoscopic mucosal resection, and endoscopic submucosal dissection. Unlike DBE, however, EDGE/GATE is often performed as a 2-stage intervention, requires creation of a fistulous tract into the RS, which can cause weight regain and persist despite attempts at endoscopic closure, and carries the risk of stent migration, which can necessitate additional procedures. Although it depends on local expertise, EDGE/GATE, therefore, seems to be a better option when repeated, prolonged access to the RS is required, or there is the need for advanced therapeutics, whereas DBE appears to be a superior option for an initial diagnostic assessment or when the expected therapeutic maneuvers can be accomplished with devices that can be used through a balloon enteroscope.

Our study benefits from including a relatively large cohort drawn from 3 sites spanning diverse geographies across the United States. We do not feel as though the inclusion of only academic referral centers limits our findings because DBE is most often performed in centers like ours. Although its retrospective nature is a limitation, we do not feel as though this diminishes the applicability of our findings to understanding the success rate and expected outcomes of these cases in a real-world clinical setting.

## Conclusions

DBE allows for safe exploration of the RS in a majority of patients when a luminal evaluation is indicated based on symptoms, imaging, or past endoscopic findings.

## Patient Consent

Given the time span of the procedures included in this study, the number of patients involved across multiple medical centers and the fact that this is a retrospective analysis, individual consent for inclusion could not be feasibly obtained from the patients included in this study. This lack of consent was approved by the Institutional Review Board at each medical center involved in this study, and all data collected were free of protected health information.

## Disclosure

All authors disclosed no financial relationships.

## Figures and Tables

**Supplementary Table 1:** Clinical findings associated with time from RYGB to DBE

Characteristic	Time between RYGB and DBE metric, years	Characteristic response		*P* value∗
		No	Yes	
Adenocarcinoma	n	70	4	.01
	Median (IQR)	13 (4-16)	22 (18-24)	
NET	n	72	1	.33
	Median (IQR)	13 (5-17)	4 (4-4)	
*H pylori*	n	71	2	.20
	Median (IQR)	13 (5-17)	7 (1-12)	
HP negative gastritis	n	62	11	.24
	Median (IQR)	14 (6-17)	7 (3-15)	
Pathology not taken	n	36	37	.28
	Median (IQR)	12 (4-16)	14 (8-17)	
Normal	n	70	3	.83
	Median (IQR)	13 (5-16)	15 (2-19)	

*DBE*, Double-balloon enteroscopy; *HP, H Pylori*, *Helicobacter pylori*; *IQR*; interquartile range; *NET*, neuroendocrine tumor; *RYGB*, Roux-en-Y gastric bypass.

Note: Time from RYGB to DBE was not available for all patients, and thus, n values may not align with Table 1.

∗ Wilcoxon rank sum test.
